# Phosphodiesterase inhibition mediates matrix metalloproteinase activity and the level of collagen degradation fragments in a liver fibrosis *ex vivo* rat model

**DOI:** 10.1186/1756-0500-5-686

**Published:** 2012-12-18

**Authors:** Sanne Skovgård Veidal, Mette Juul Nielsen, Diana Julie Leeming, Morten Asser Karsdal

**Affiliations:** 1 Nordic Bioscience A/S, Herlev Hovedgade 207, 2730, Herlev, Denmark

**Keywords:** Precision-cut liver slices, Fibrosis, *Ex vivo*, cAMP

## Abstract

**Background:**

Accumulation of extracellular matrix (ECM) and increased matrix metalloproteinase (MMP) activity are hallmarks of liver fibrosis. The aim of the present study was to develop a model of liver fibrosis combining *ex vivo* tissue culture of livers from CCl_4_ treated animals with an ELISA detecting a fragment of type III collagen generated *in vitro* by MMP-9 (C3M), known to be associated with liver fibrosis and to investigate cAMP modulation of MMP activity and liver tissue turnover in this model.

**Findings:**

*In vivo:* Rats were treated for 8 weeks with CCl_4_/Intralipid. Liver slices were cultured for 48 hours. Levels of C3M were determined in the supernatants of slices cultured without treatment, treated with GM6001 (positive control) or treated with IBMX (phosphodiesterase inhibitor). Enzymatic activity of MMP-2 and MMP-9 were studied by gelatin zymography. *Ex vivo:* The levels of serum C3M increased 77% in the CCl_4_-treated rats at week 8 (p < 0.01); Levels of C3M increased significantly by 100% in fibrotic liver slices compared to controls after 48 hrs (p < 0.01). By adding GM6001 or IBMX to the media, C3M was restored to control levels. Gelatin zymography demonstrated CCl_4_-treated animals had highly increased MMP-9, but not MMP-2 activity, compared to slices derived from control animals.

**Conclusions:**

We have combined an *ex vivo* model of liver fibrosis with measurement of a biochemical marker of collagen degradation in the condition medium. This technology may be used to evaluate the molecular process leading to structural fibrotic changes, as collagen species are the predominant structural part of fibrosis. These data suggest that modulation of cAMP may play a role in regulation of collagen degradation associated with liver fibrosis.

## Findings

### Introduction

Liver fibrosis due to viral or alcohol-induced injury is one of the leading causes of death worldwide [1]. Liver biopsy is the most commonly used method for fibrosis assessment, but it is invasive, associated with patient discomfort and, in rare cases, with serious complications [2-4]. Therefore, research has focused on the evaluation of non-invasive methods for the assessment of liver fibrosis [5], including a highly enforced effort in discovering and developing biochemical markers for liver fibrosis assessment. In alignment there is an urgent need for applied translational science, in which information from preclinical settings may be translated to clinical settings. One such approach may be to investigate the same biochemical analyte in liver explants as a model of liver fibrosis, in animal models of liver fibrosis, and then finally in clinical settings.

Fibrosis may be described as extensive scar formation, observed as increased deposition and abnormal distribution of extracellular matrix (ECM) components such as collagens and proteoglycans. ECM remodeling is a key process of tissue homoeostasis [6-8], and specific proteolytic activities are a prerequisite for a range of cellular functions and interactions during the process [9]. The specific proteolytic activities are precisely coordinated under physiological situations, with a specified sequence of events resulting in controlled tissue turnover. In pathological situations, including inflammation, fibrosis and cancer, the normal damage/repair balance is displaced [10], leading to excessive remodeling. As a consequence of this tissue turnover, there is a release of several protein degradation fragments specific for the combination of the involved proteases, the affected organ and the disease. The fragmentation results in exposure of new peptide ends (so-called neo-epitopes) to which specific antibodies can be developed. These neo-epitopes may be used for the design of molecular biochemical markers [11].

In the healthy human liver the most abundant collagens are the fibril-forming type I and type III [5]. Fibril-forming collagens are synthesized as precursor molecules with large propeptide extensions at both the N- and C-terminals of the molecule [12]. The mature propeptides are cleaved from procollagen by N- or C-terminal proteinases, and mature collagen is integrated into the ECM [12,13]. During fibrogenesis, type I and III collagen levels increase up to 8 times [14]. Endopeptidases such as matrix metalloproteinases (MMPs) play a major part in the degradation of extracellular macromolecules such as collagens and during fibrogenesis the levels of MMPs increase. With respect to excessive proteolytic activity in the fibrous tissue, the gelatinases MMP-2 and MMP-9 have been investigated and documented to be highly regulated [15-17]. Thereby a fragment of type III collagen generated *in vitro* by MMP-2 and MMP-9 may be a biochemical marker for liver fibrosis. Thus by analysis of cleavage fragments generated by MMP-9 of type III collagen, and the development of a specific assay quantifying a validated fragment, novel tools with increased sensitivity and specificity for some types of fibrosis may be developed. We have previously identified fragments of other collagens, and developed assays for those [18-20], and very recently identified a unique type III collagen degradation product (C3M), *in vitro* generated by MMP-2 and MMP-9. We consequently developed a novel Enzyme-linked immunosorbent assay (ELISA) using monoclonal antibody to detect this specific fragment, which later on demonstrated to be highly associated with liver fibrosis [21-24].

Several animal models for liver fibrosis have been developed, most of them in small rodents [25], each with individual strengths and weaknesses. These different rodent models are complementary as they represent different pathways to fibrosis, as also seen in human disease. Bile duct ligation (BDL) in rats has been used as a model of chronic liver injury due to its resemblance to hepatocyte damage, hepatic stellate cell (HSC) activation, and liver fibrosis observed in human cholestatic liver diseases [1,25]. Carbon tetrachloride (CCl_4_) is a hepatotoxin that causes acute liver injury and, when given repetitively at a low dose, induces liver fibrosis. It is a highly reproducible and robust model which is used to resemble alcoholic and non-alcoholic steatohepatitis with the consequent fibrosis and cirrhosis in humans [1,25].

*Ex vivo* models enables the study of complicated processes in a multicellular system in which cell-cell and cell-matrix interactions are maintained. The ECM holds various components, both inhibitors and promoters of cell function, which are absent in traditional plastic culture systems [26,27]. In liver fibrosis where tissue turnover plays a fundamental role, the ECM has both structural and biochemical features, which are not easily accounted for by either hepatic stellate cell line (e.g. HSC-T6) or isolation of primary HSC [28]. A potential *ex vivo* model for studying liver fibrosis (and HSC activation) are the precision-cut liver slices (PCLS). Several researches have investigated and used the liver explant model, first reported by Otto Warburg in 1923 [29]. Since then, these experiments have for the major part been performed in healthy livers. Thus in the current study, we employed the CCl_4_ model, with disease affected livers, and cultured these under optimal condition compared to healthy control liver, assessed by the novel liver fibrosis marker C3M, to develop an *ex vivo* model system for investigation of the processes involved in liver fibrosis tissue turnover.

Induction of cyclic AMP (cAMP) by pan-specific phosphodiesterase (PDE) inhibition has been shown to modulate MMP activity in a cartilage *ex vivo* models [30]. These studies clearly suggested that cAMP induction was essential for MMP activity and tissue turnover, both processes that are highly regulated in fibrotic diseases. We aimed at testing the hypothesis whether cAMP induction may in addition to cartilage turnover also be important for tissue turnover associated with liver fibrosis, and thus a more common regulator of MMP mediated tissue turnover.

In the present study, we aimed at using the novel molecular biochemical marker C3M to 1) develop an *ex vivo* model in which the same biochemical marker may be measured *ex vivo*, in animal settings and clinical setting to enable translational research and 2) to use this model to preliminary investigate modulation of MMP activity and liver tissue turnover by PDE inhibition.

### Materials and methods

#### Reagents

All reagents were standard high-quality chemicals from companies such as Merck and Sigma-Aldrich. Krebs-Heinseleit buffer used to preparation of PCLS was from Amplicon (Skovlunde, DK). The culture medium Williams medium E was from Lonza (Verviers, BG). IBMX was from Sigma (Poole, UK), while GM6001 was from AM Scientific (Pleasant Hill, CA). AlamarBlue assay was from AbD Serotec (Oxford, UK).

#### Animal experiment

20 male Spraque-Dawley rats aged 4 months were housed at the animal facilities at Nordic Bioscience, Denmark. The experiments were approved by the Animal Ethics Committee of the Danish Ministry of Justice (2008/561-1450). The rats were housed in standard cages, with bedding and nest material at 18-22°C and fed with standard pellet diet and tap water ad libitum. The rats were kept under conditions of a 12 hr light/dark cycle. Liver fibrosis was induced by intraperitoneal injections (i.p.) of CCl_4_ twice a week for 8 weeks starting one week after acclimatization. The rats were divided in two groups, 14 rats were treated with 10% CCl_4_ in intralipid (0.5 ml/kg) and 6 vehicle rats were treated with intralipid (0.5 ml/kg).

#### Blood sampling

Blood samples at baseline and termination were taken under inhalation anesthesia using isoflurane from the retro-orbital sinus of rats which had fasted for at least 14 hr. The blood were left for 30 min at room temperature to clot and centrifuged at 3000 rpm for 10 min. Liquid were transferred to new tubes and centrifuged again at 3000 rpm for 10 min. Serum were transferred into clean tubes and stored for −20°C until use.

#### Tissue handling

After the rats were euthanized, the liver was carefully excised and weighted. A small part of one of the lobes was fixated in 4% formaldehyde for at least 24 hr and embedded in paraffin. Sections were cut 4–5 μm thick, mounted on glass slides and stained with 1% Sirius Red F3B (Sigma-Aldrich, St. Louis, MO) in saturated picric acid (Sigma-Aldrich). The liver sections were evaluated by histological changes in tissue architecture, presence of inflammation and proliferation of liver fibrosis.

#### Immunohistochemistry

Liver sections (4–5 μm) were de-paraffinised, hydrated and further peroxidase activity was blocked with the addition of 0.4% hydrogen peroxide. Sections were the incubated with a polyclonal antibody against α-SMA (1:400, Abcam, Cambridge, UK). Sections were then rinsed and the antibody binding was depicted using the Super Sensitive Polymer-HRP IHC Detection System combined with AEC substrate, according to the supplier’s instructions (Biogenex, Taby, Sweden). Sections were counterstained with Mayer’s haematoxylin. Digital photographs were taken using an Olympus Bx60 microscope with x40 magnification and an Olympus 5050-zoom digital camera (Olympus, Tokyo, Japan).

#### Ex vivo experiments

For *ex vivo* liver tissue culture experiments, fibrosis was induced as described. Rat livers were excised from adult male Spraque-Dawley rats, weighted, stored in cold PBS and liver fibrosis was evaluated by gross appearance. PCLS were prepared from the livers in ice-cold Krebs-Heinseleit buffer containing 25 mM glucose, 10 mM HEPES and 25 mM NaHCO_3_, and sliced on the TSE Krumdieck tissue slicer MD 4000 as previously described [31]. The PCLS were cultured for 2 hr at 37°C under carbogen atmosphere in sterile 48 well plates containing 300 μl Williams Medium E containing 25 mM glucose, 10 mg/ml gentamycin and 10% fetal bovine serum (FBS). After 2 hr the serum media was removed and the slices were washed in serum-free media. New serum-free media was added containing the respective treatments and the PCLS were cultured for 48 hr. Treatments used in the experiments were 100 μM IBMX, a non-specific PDE inhibitor, and 10 μM GM6001, a MMP inhibitor. After the culturing period supernatants were collected and stored at −20°C until use.

#### Cell viability measured by AlamarBlue

AlamarBlue is a sterile non-toxic, aqueous oxidation-reduction indicator that yields colorimetric changes and a fluorescent signal in response to metabolic activity. For the quantification of cell viability, we used the AlamarBlue assay. The active compound, resazurin, is an oxidation-reduction indicator that changes from the oxidized non-fluorescent form (indigo) to the reduced fluorescent form (purple) according to the viability and proliferating activity of cells [32]. In short, 300 μl 10% AlamarBlue was added to each well of the microtiter plate containing PCLS and incubated for 2 hr at 37°C while shaking. The results were measured by ELISA reader at 540–590 nm.

#### Gelatinase zymography

Activity and expression of MMP-2 and MMP-9 were investigated using gelatinase zymography. Supernatants from the *ex vivo* experiment were diluted 3:4 in 4x sample buffer, heated at 37°C for 15 min and applied for gel-electrophoresis on 10% SDS-polyacrylamide gel using the substrate 3 mg/ml gelatin. After electrophoresis, gels were washed three times before four days incubation at 37°C with gentle agitation in 1% Triton-X, 100 mM Tris Base, 13 mM CaCl_2_, 0.2 mM ZnCl_2_, 6 mM NaN_3_, and pH 7.5. Gels were stained for 15 min with a solution containing 0.25% Coomassie R-250 with 45% methanol and 3% acetic acid. The gels were destained in a solution of 20% methanol, 17% ethanol, 7% acetic acid, and 0.6% diethyl-ether for 45 min until clear bands were obtained. Gels were finally soaked in 2% glycerol, dried in plastic bags and scanned for documentation. The intensity of the bands was quantified by densitometry analysis shown as average pixel number.

#### C3M ELISA

The ELISA was performed as previously described [21-24].

#### Statistical analyses

Results are shown as mean ± standard error of mean (SEM). Five vehicle rat livers and 9 CCl_4_ treated rat livers were used in the *ex vivo* experiment with 10 replicas for each treatment. Differences between mean values were compared by nonparametric Mann-Whitney’s t-test for two-tailed observations. All statistical analyses were performed in GraphPad Prism software v.5 (GraphPad Software, San Diego, CA). P values less than 0.05 were considered significant.

### Results

#### Evaluation of in vivo experiment

**Figure 1:**
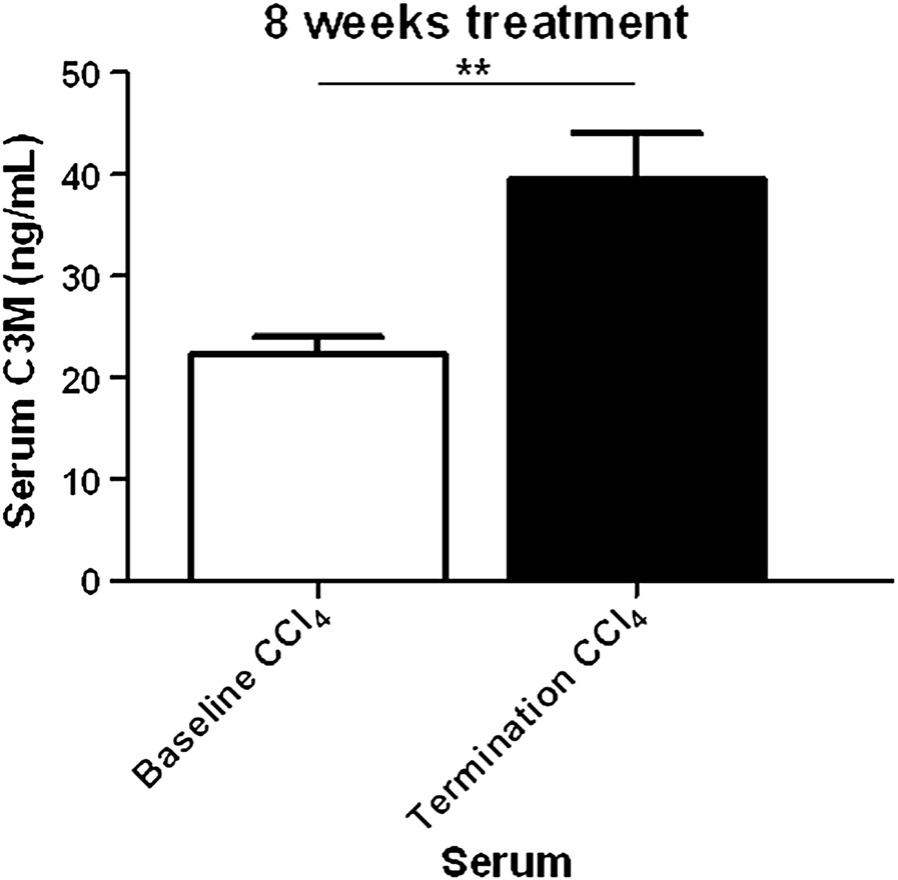
**Serum levels of C3M in CCl**_**4 **_**and Vehicle rats at baseline compared to termination after 8 weeks of treatment. **Number of rats used (N): Vehicle N = 5, CCl_4 _N = 11. Asterisks **p < 0.01.

**Figure 2:**
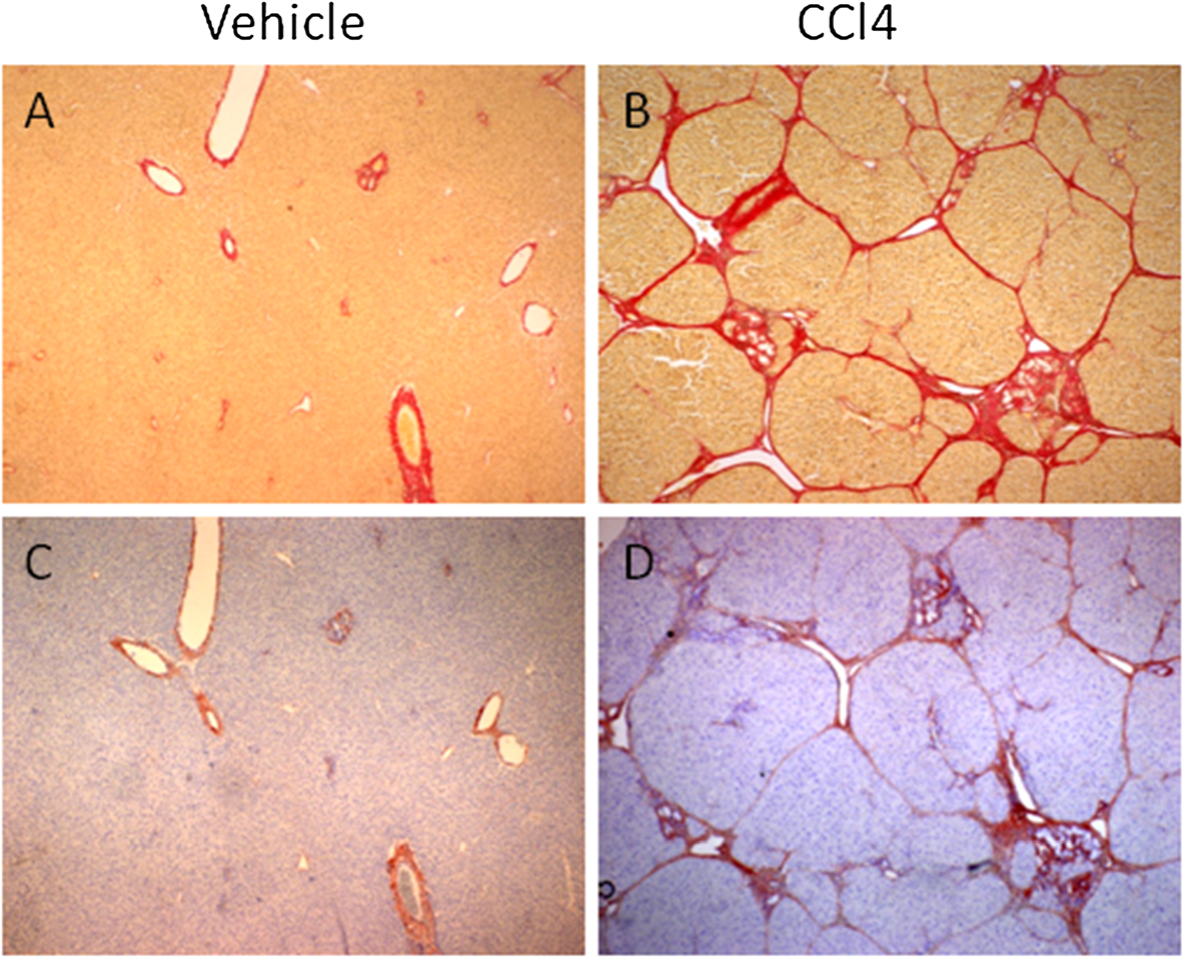
**Sirius Red staining of hepatic structure in vehicle rats (A) compared to CCl**_**4 **_**treated rats (B) after 8 weeks of treatment. **The hepatic tissue architecture around the portal tracts is disrupted in fibrotic livers compared to vehicle and the amount of collagen is increased. **Immunohistochemical analysis of α-SMA in vehicle rats (C) and CCl4 treated rats** (**D**). α-SMA is localized around the fibrotic bands. Original magnification ×40.

Three CCl_4_ animals died during the experiment. Serum C3M levels were increased with 77% from baseline to termination in CCl_4_ treated rats (p = 0.0025) (Figure 1). Histological examination of the livers from control rats showed no sign of fibrosis as the tissue architecture appeared normal (Figure 2A), while the livers from CCl_4_ treated rats showed increased collagen deposition around the portal tracts and fibrotic septae (Figure 2B). By immunohistochemistry, α-SMA deposition was found exclusively in the venous wall of control rats (Figure 2C). In contrast, in CCl_4_ treated rats α-SMA was located along the fibrotic bands (Figure 2D).

#### Ex vivo tissue culture

**Figure 3:**
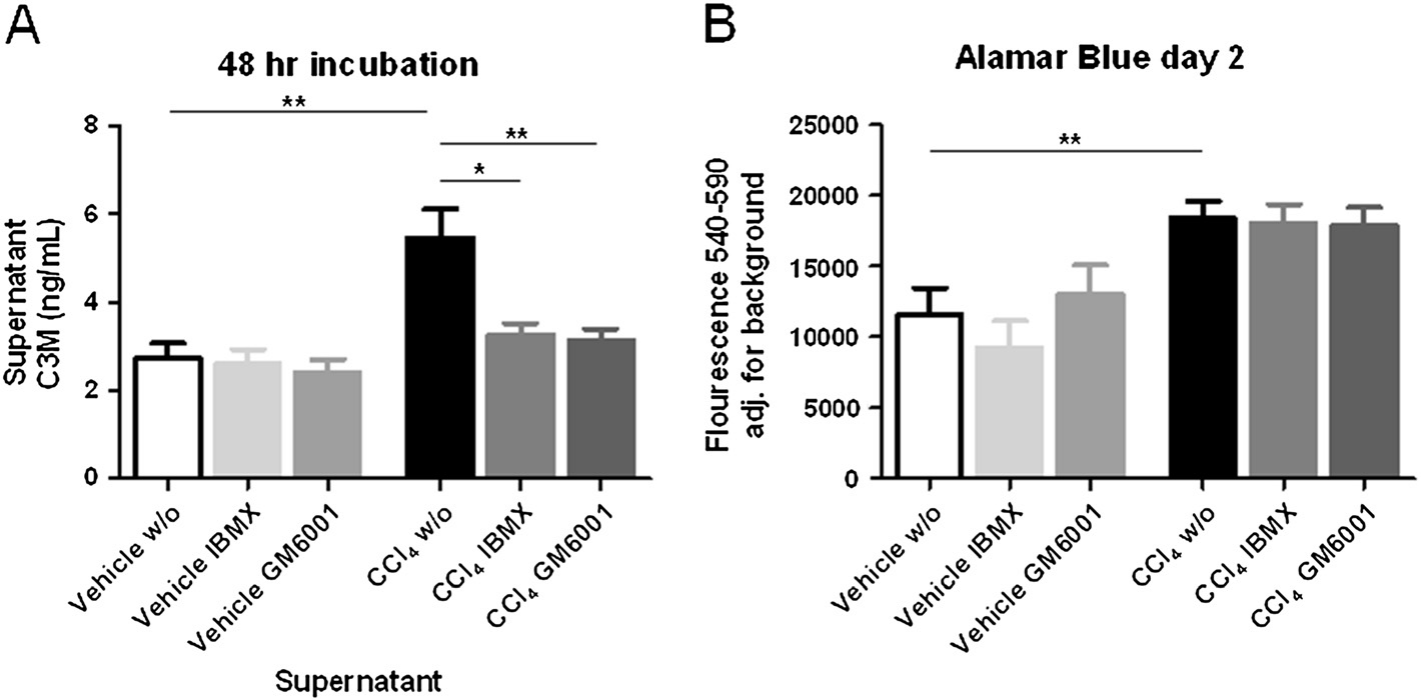
**A: C3M release from *ex vivo *cultures after 48 hr incubation. **Liver slices from fibrotic or vehicle rats were cultured in Williams E medium and released C3M levels in the supernatants were measured in the C3M ELISA. W/o: Williams E medium without any intervention; IBMX: Williams E medium + 100 μM IBMX; GM6001: Williams E medium + 10 μM GM6001. Number of livers used (N): Vehicle N = 5 fibrotic N = 9. Number of liver slices used pr liver (n): n = 10 in each condition. Asterisks *p < 0.05, **p < 0.01. **B: AlamarBlue was investigated as a measure of metabolic activity in fibrotic and vehicle liver slices with addition of IBMX or GM6001 in Williams E medium after 48 hour culturing period.** W/o: Williams E medium without any intervention; IBMX: Williams E medium + 100 μM IBMX; GM6001: Williams E medium + 10 μM GM6001. Number of livers used (N): Vehicle N = 5, Fibrotic N = 9. Number of liver slices used pr liver (n): n = 10 in each condition. Asterisks **p < 0.01.

Normal and fibrotic livers were dissected and precision-cut for *ex vivo* cultures, following *ex vivo* slices were cultured for 48 hr. Samples from each liver were divided in three groups, i.e. w/o (without any intervention), IBMX (100 μM IBMX was added to the culture medium), and GM6001 (10 μM GM6001 was added to the culture medium); n = 10 for all conditions. The supernatants were collected and measured in the C3M ELISA, measuring *in vitro* generated MMP-9 fragments of type III collagen. There was a 100% increase (p = 0.0018) in supernatants of fibrotic liver slices without any intervention compared to vehicle samples (Figure 3A). Treatment with IBMX or GM6001 had no effect on the vehicle samples. When exposing the fibrotic liver slices to IBMX, the level of C3M were reversed back to the level of vehicle samples (p = 0.0348). Adding GM6001 to the fibrotic liver slices served as a positive control as it inhibits MMP activity (p = 0.0077).

AlamarBlue was investigated as a measure for metabolic activity (Figure 3B). A 60% increase was observed in fibrotic liver slices compared to vehicle without intervention (p = 0.035). Adding IBMX or GM6001 to the fibrotic liver slices had no effect on metabolic activity.

**Figure 4:**
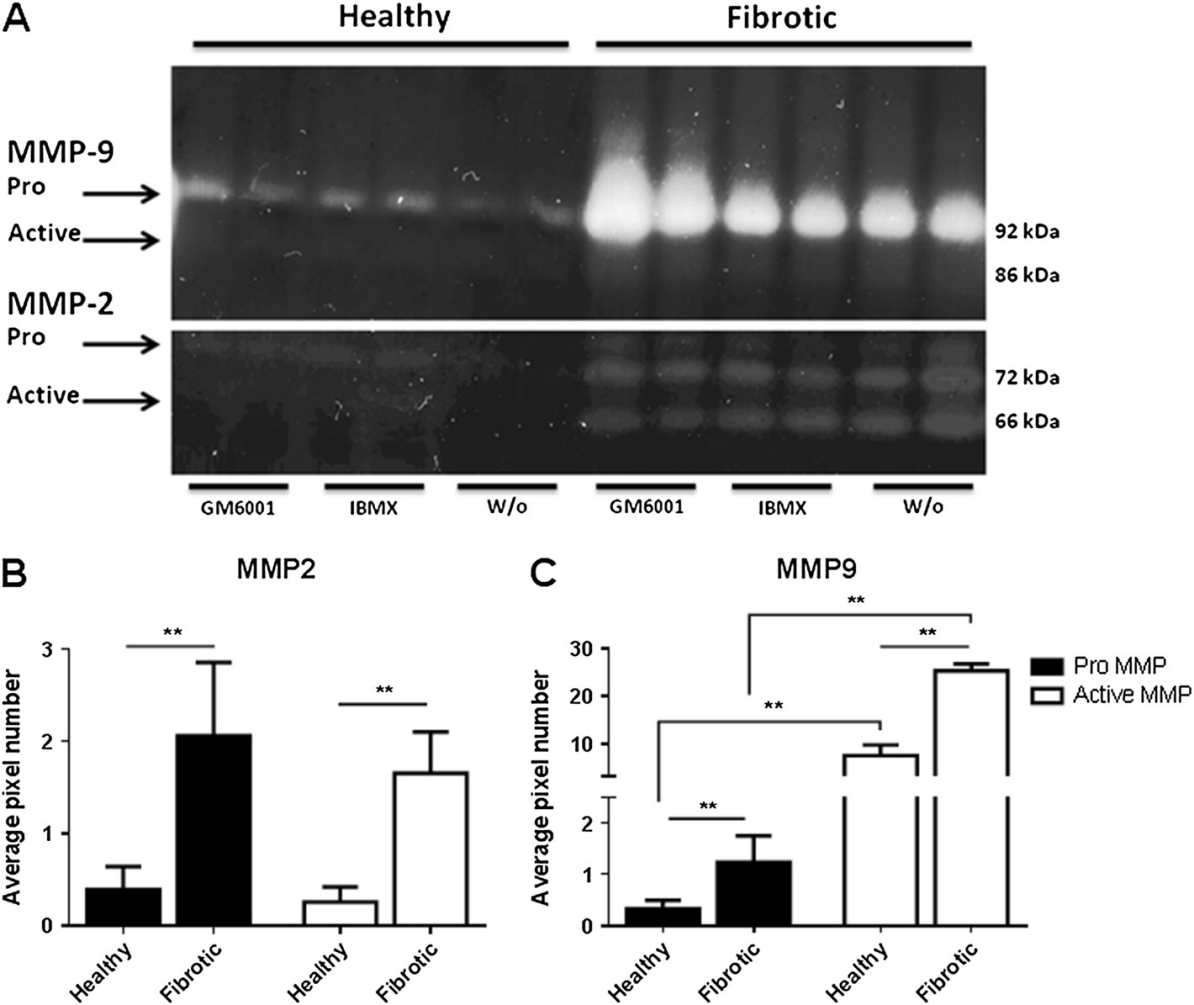
**A: MMP expression and activity assessment by gelatin zymography. **Liver explants were cultured without intervention (w/o) or with either 100 μM IBMX or 10 μM GM6001. The MMP activity in the supernatants after 48 hr is identified on the zymography gel by the standards for MMP-2 and MMP-9. Pro-MMP-9 and active MMP-9 are seen as bands migrating 92 kDa and 86 kDa, respectively, while pro-MMP-2 and active MMP-2 are seen as bands migrating 72 kDa and 66 kDa, respectively. **B-C: Quantification of MMP-2 and MMP-9 activity in supernatants from healthy and fibrotic liver slices assessed by densitometry. **Asterisks **p < 0.01.

Gelatin zymography revealed that MMP-2 and MMP-9 activity were clearly increased in supernatants from fibrotic liver slices compared to control. The MMP-9 activity was to a lesser extent increased when treated with GM6001 compared to liver slices without any intervention in the supernatants from fibrotic liver slices. A lower decrease in MMP-9 activity in supernatants treated with IBMX was also observed (Figure 4A). Densitometry analysis of the bands on the zymography revealed a significant increase in pro- and active MMP-2 (p = 0.0022 and p = 0.0022, respectively) as well as in pro- and active MMP-9 (p = 0.0022 and p = 0.0022, respectively) in fibrotic liver slices compared to controls (Figure 4B-C). Furthermore, there was a significant increase in active MMP-9 compared to pro-MMP-9 in supernatants from both healthy and fibrotic liver slices (p = 0.0022) (Figure 4C).

### Discussion

Liver injury is a multifactor process, involving many cell types, mediators and cell-cell and cell-matrix interactions. The main advantages of the PCLS model are that the cells are the maintained in the 3-dimensional organ structure, with the same relative cell number and orientation towards other cells [33], which makes the model a valuable tool for studying tissue turnover, due to the high *in vivo* likeness [34]. The PCLS model has been applied recently to the study of HSC activation [31], and in addition it has also been shown that endothelial cells [35] and Kupffer cells [36] remain viable in these cultures. Especially for studying ECM remodeling in the liver during fibrosis, the PCLS model provide several advantages that seem essential [37].

This study provides the first measurement of a liver fibrosis marker in *ex vivo* cultures that in other settings have been shown to correlate to the extent of liver fibrosis *in vivo.* Furthermore, we used a PDE inhibitor, that in other settings have been shown to modulate MMP activity [30], to demonstrate that this *ex vivo* liver fibrosis model in combination with the biochemical marker may provide further insights into ECM remodeling in the liver during pathology. Hopefully this will enable other researchers to investigate and validate key processes in liver fibrosis and enable more developments of treatments for liver diseases.

The levels of the C3M fragment was 100% elevated in the *ex vivo* cultures of fibrotic liver slices. In the present study we used the general MMP inhibitor GM6001 as a positive control [27]. Following exposure of the liver explants to GM6001 the level of the C3M was restored to normal levels, suggesting that this marker is both specific for MMP activity and pathology relevant. Furthermore, in the *in vivo* experiment a 77% increase of serum C3M fragment in CCl_4_ rats at termination compared to baseline was observed. The two experiments combined illustrate the accuracy for this marker both *in vivo* and *ex vivo*. Additionally, our data are in alignment with previous investigation of C3M as a marker for liver fibrosis, which demonstrated C3M was elevated in serum from a BDL rat model [23,38] and a CCl_4_ inhalation model [39] respectively.

cAMP modulation has in other settings been shown to modulate MMP activity [30,40,41]. In particular in *ex vivo* models, cAMP induction by either the PDE inhibitor IBMX or induction of adenylate cyclase by forskolin, resulted in inhibition of MMP expression and activity [30]. A similar pattern was seen in the current studies, suggesting that 1) cAMP is a general regulator of MMP activity and 2) that this model and biomarker may be used for important hypothesis testing and aid in the understanding of the processes of ECM remodeling in the liver during pathology. One drawback of the study is lack of intracellular cAMP measurement, thus we can only hypothesize that inhibition of PDE modulate cAMP levels as previously reported [30]. Further research is needed to understand the effect of the PDE inhibition in hepatocellular models. The current findings suggest a possibly path of investigation in terms of using the described tools allowing for a deeper molecular understanding of fibrogenesis and fibrolysis.

Interesting, inhibition of MMP activity seemed to increase the MMP-2 and MMP-9 expressions, suggesting a regulatory feedback loop which augments MMP production in the absence of MMP activity. Such a feedback loop have previously been reported for protease inhibition, and suggest further caution when targeting protease inhibition on various diseases [27,42]. Whether MMP-2 and MMP-9 play different roles in regulating fibrosis has not yet been elucidated; however it is generally accepted that both are involved in fibrogenesis and fibrosis resolution [43-45]. Gelatin zymography performed with acute liver injury studies showed an over-expression of both MMP-2 and MMP-9 in BDL rats compared to sham [46], while others have shown that only MMP-2 was up-regulated in CCl_4_ rats [47]. In the current experiment, MMP-9 was highly expressed compared to MMP-2, in fibrotic livers. When interpreting the levels of MMP-2 and MMP-9, it is important to take into account the levels of Tissue Inhibitor of Matrix Metalloproteinase (TIMP) for a more complete picture of the proteolytic capacity [15]. Further studies are needed to investigate this balance in different models of liver fibrosis, to understand the molecular action of each of the individual players that might be highly stage dependent [48,49]. There was no clear inhibition of MMP activity in the presence of IBMX, albeit a significant lower release of the C3M fragment was demonstrated. The measurement of MMP-2 and MMP-9 is done by zymography in the conditioned medium. There may be a range of differences in the matrix of the fibrotic liver, and in that local activation milieu, compared to that of the “simple” conditioned medium. Thus, the cascade of activation proteases and generating the C3M fragment is dependent on MMP activity, and when only one single protease is present on MMP-9 activity. However, while in a more complex environment multiple protease are present, and both activation cascades and local positioning is complex and may be involved, while general MMP activity still is essential for generating the C3M fragment. This suggests that other MMPs are responsible for generation of this fragment than MMP-2 and MMP-9, and that other MMPs may have been attenuated. Thorough characterization of C3M is ongoing in our group. Investigation of *in vitro* cleaved type III collagen with ADAM-TS’s, MMP-1, MMP-3, MMP-7, MMP-12, and MMP-13 showed no reactivity using the C3M antibody (data not shown). However, further biological characterization is needed in order to understand the generation of the C3M fragment *in vivo* and *ex vivo.*

In the present study AlamarBlue was used for quantitative measurements of cell viability and proliferation. This dye has previously been undertaken in other settings by other researchers [50]. Interestingly we found a 60% higher metabolic activity in fibrotic liver slices compared to vehicle, suggesting that the liver toxicity by CCl_4_ induced high activity within the liver tissue. This clearly demonstrated the grave pathological induction in the tissue by CCl_4_ that may be part of the processes leading to liver fibrosis. Furthermore, the cell viability remained the same as for fibrotic rat liver slices after addition of IBMX and GM6001 suggesting that the decreased levels of the C3M fragment released into the supernatants is not caused by compound toxicity but rather altered MMP activity. This phenomenon can be explained by the increased proliferation and ECM deposition by activated HSCs in liver fibrosis. Furthermore, during fibrogenesis hepatocytes undergo apoptosis thus releasing intracellular substances. Mitochondrial, cytosolic, and microsomal enzymes have been shown to reduce Alamar Blue [51], which might explain the increase in Alamar Blue activity in fibrotic liver slices compared to vehicle liver slices. Further studies are needed to understand this high metabolic activity in details.

The C3M assay was designed with that purpose, as the epitope is a conserved sequence through many species, and has been identified in: human, monkey, rat, mouse, and sheep. We used the C3M assay in order to investigate the type III collagen degradation in both *ex vivo* and *in vivo* model of liver fibrosis. We found a highly significant, and more than 100% fold up regulation of the neo-epitope in fibrotic liver tissue compared to the vehicle tissue in the *ex vivo* liver tissue cultures.

In the current experiments we used fibrotic livers, suggesting that the model may reflect parts of the processes of ECM remodeling in liver pathology. In alignment, as fibrotic livers represent a highly altered metabolic profile with activated cellular phenotypes [31,36,52], such systems may allow for improved translational research, compared to that of healthy livers, allowing a more predictive outcome. Further research is needed to understand whether this model may both be used for investigation of the processes involved in liver fibrogenesis as well as those processes involved in liver fibrosis resolution.

In conclusion, we have developed a novel *ex vivo* model system with pathological affected livers in a combination with a biochemical marker that is the result of extensive matrix remodeling associated with fibrosis. We found a highly significant, and more than 100% fold up regulation of the neo-epitope in fibrotic liver tissue compared to the vehicle tissue in the *ex vivo* liver tissue cultures. Following exposure of the liver explants to GM6001 or IBMX, C3M levels was restored to control levels. Gelatin zymography demonstrated CCl_4_-treated animals had highly increased MMP-9, but not MMP-2 activity, compared to slices derived from control animals. These data suggest that modulation of cAMP through PDE inhibition may play a role in the regulation of collagen turnover during liver fibrosis. Further studies are needed to proof this hypothesis, such as induction of adenylate cyclase by forskolin. As the biochemical marker C3M can be assessed in both *ex vivo* and preclinical, this may allow for better translational research assisting drug discovery and development in the liver fibrosis field.

## Abbreviations

ADAMTS: A Disintegrin and metalloproteinase with trombospondin motifs; BDL: Bile duct ligation; cAMP: Cyclic AMP; CCl_4_: Carbon tetrachloride; ECM: Extracellular matrix; ELISA: Enzyme-linked immunosorbent assay; FBS: Fetal bovine serum; FDA: Food and drug administration; HSC: Hepatic stellate cell; i.p.: Intraperitoneal injection; IBMX: 3-isobutyl-1-methylxanthine; MMP: Matrix metalloproteinase; PCLS: Precision-cut liver slices; PDE: Phosphodiesterase; SEM: Standard error of mean; TIMP: Tissue inhibitor of matrix metalloproteinase.

## Competing interests

All authors are full-time employees at Nordic Bioscience. Morten A. Karsdal holds stocks in Nordic Bioscience A/S.

## Authors’ contributions

SSV prepared sections for the manuscript, designed and guided the *in vivo* and *ex vivo* experiments. MJN prepared sections for the manuscript, designed and guided the *in vivo* experiment and did the *ex vivo* experiments. DJL prepared sections for the manuscript. MAK prepared and guided the manuscript. All authors read and approved the final manuscript.
